# RhoA Regulates Peroxisome Association to Microtubules and the Actin Cytoskeleton

**DOI:** 10.1371/journal.pone.0013886

**Published:** 2010-11-08

**Authors:** Lukas Schollenberger, Thomas Gronemeyer, Christoph M. Huber, Dorothee Lay, Sebastian Wiese, Helmut E. Meyer, Bettina Warscheid, Rainer Saffrich, Johan Peränen, Karin Gorgas, Wilhelm W. Just

**Affiliations:** 1 Heidelberg Center of Biochemistry, University of Heidelberg, Heidelberg, Germany; 2 Medical Proteom-Center, University of Bochum, Bochum, Germany; 3 Department for Molecular Genetics and Cell Biology, University of Ulm, Ulm, Germany; 4 Department of Internal Medicine V, University of Heidelberg, Heidelberg, Germany; 5 Institute of Biotechnology, University of Helsinki, Finland; 6 Department of Anatomy and Medical Cell Biology, University of Heidelberg, Heidelberg, Germany; University of Birmingham, United Kingdom

## Abstract

The current view of peroxisome inheritance provides for the formation of new peroxisomes by both budding from the endoplasmic reticulum and autonomous division. Here we investigate peroxisome-cytoskeleton interactions and show by proteomics, biochemical and immunofluorescence analyses that actin, non-muscle myosin IIA (NMM IIA), RhoA, Rho kinase II (ROCKII) and Rab8 associate with peroxisomes. Our data provide evidence that (i) RhoA in its inactive state, maintained for example by *C. botulinum* toxin exoenzyme C3, dissociates from peroxisomes enabling microtubule-based peroxisomal movements and (ii) dominant-active RhoA targets to peroxisomes, uncouples the organelles from microtubules and favors Rho kinase recruitment to peroxisomes. We suggest that ROCKII activates NMM IIA mediating local peroxisomal constrictions. Although our understanding of peroxisome-cytoskeleton interactions is still incomplete, a picture is emerging demonstrating alternate RhoA-dependent association of peroxisomes to the microtubular and actin cytoskeleton. Whereas association of peroxisomes to microtubules clearly serves bidirectional, long-range saltatory movements, peroxisome-acto-myosin interactions may support biogenetic functions balancing peroxisome size, shape, number, and clustering.

## Introduction

Mammalian peroxisomes associate with the microtubular cytoskeleton for intracellular transport [1], [2]. Three distinct states of motility were recognized, long-range saltations, oscillations and arrest. Furthermore, we demonstrated that peroxisome motility is subject to regulation by extracellular ATP-lysophosphatidic acid (LPA) receptor co-stimulation. Signaling involves trimeric G_i_/G_o_ protein, PLC, Ca^2+^ influx, cPKC, MAP kinase and PLA_2_ and mediates peroxisomal arrest [3]–[5]. Via G_12/13_ the LPA receptor activates the Rho pathway preventing long-range peroxisomal motility that seems to be regulated by a complex signaling network [4], [6].

These studies did not show the involvement of the actin cytoskeleton in the motility of mammalian peroxisomes. However, plant and yeast peroxisomes associate with the actin cytoskeleton for saltatory movement and inheritance, respectively. In plant cells interference with the actin cytoskeleton results in loss of saltatory movement, aggregation, and complete cessation of peroxisome motility [7]. In *S.cerevisiae* peroxisomes are targeted and segregated to the developing bud by a highly ordered process involving actin and Myo2p, a class V myosin motor protein [8], [9]. Actin-based movement of mammalian organelles other than peroxisomes has however been demonstrated [10], [11]. Two types of motility are described. One is based on actin polymerization itself propelling organelles by an “actin comet tail” toward the cell center. This type has been described for phagosomes and macropinosomes and may also be involved in transport between endosomes and lysosomes [12], [13]. The other type of movement depends on actin-based myosin motors and utilizes myosins of the classes I, II, V or VI. This myosin-dependent movement has been implicated in dynamics of the ER, lysosomes, Golgi-derived vesicles, secretory granules, recycling endosomes and melanosomes [14]–[16].

Some indirect evidence suggests that mammalian peroxisomes also associate with the actin cytoskeleton. Drp-1 known to be involved in mitochondrial fission in an actin cytoskeleton-dependent manner [17] was shown also to localize to peroxisomes. Over-expression of a dominant-negative mutant of Drp-1 prevents peroxisome division in a human hepatoma cell line [18]–[20]. Generally, members of the dynamin protein family are known regulators of vesicle trafficking implicated in constricting and severing membrane tubules [21].

Ultrastructural studies on peroxisome proliferation in rat liver suggest a sequential mechanism of membrane tubulation and constriction prior to fission [22], [23]. Thus, a force-generating system such as the acto-myosin complex is likely to be involved. Frequently, these processes are regulated by small GTPases known to support membrane traffic as well as the local organization of both the microtubular and the actin cytoskeleton [10], [24]. Members of the Rho, Rab and Sar1/Arf families are implicated [24], [25] and Rho1p and Arf1 recently shown to associate with peroxisomes [4], [6], [22], [23], [26], [27]. Mammalian peroxisomes in presence of GTP-primed cytosol recruit Arf1 and the COPI coat subunits and among the proteins specifically enriched with *S. cerevisiae* peroxisomes Rho1p was identified by a mass spectrometric screen.

So far our knowledge on the mechanism of peroxisome proliferation and the role small GTPases and the cytoskeleton play is rather incomplete. Peroxins, such as Pex11p, Pex25p and Pex27p are likely to be involved [28]–[30]. However, factors are still missing in order to fully understand the process. In the present study we initiated proteomics analyses to identify components that recruit onto peroxisomes from the cytosol. We found a significant number of candidate proteins some of which were further investigated. Here we report alternate binding of peroxisomes to microtubules and actin microfilaments dependent on the activation state of RhoA. Our studies indicate a sequence of events in which activated RhoA by binding to peroxisomes detaches the organelles from microtubules. Concomitantly, activated RhoA may favor peroxisome association with the actin cytoskeleton possibly by recruiting Rho kinase (ROCKII). Phosphorylation of myosin light chain induces actin-activated non-muscle myosin IIA (NMM IIA) ATPase activity enabling force generation.

## Results

### Peroxisome-microtubule association

**Figure 1:**
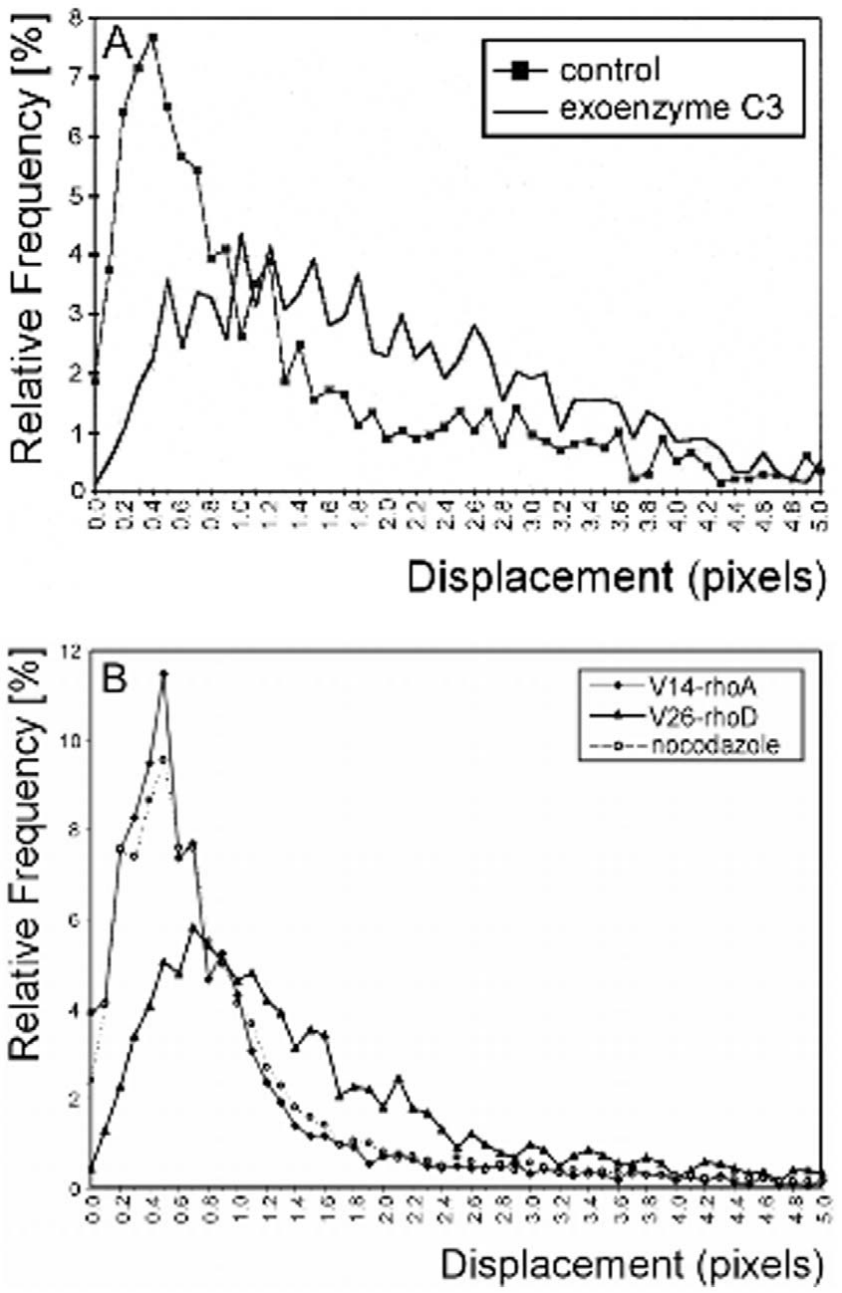
Particle-tracking analysis of GFP-SKL-marked peroxisomes in CHO cells. (A) Treatment of cells with *C. botulinum* toxin exoenzyme C3 (5 mg/ml, 24 h prior to analysis) known to inactivate RhoA significantly increases the number of peroxisomes moving long distances. (B) In cells expressing dominant-active V14-RhoA long-range movement is abolished and peroxisomal motility is indistinguishable to that in cells treated with nocodazole to depolymerize microtubules. Expression of dominant-active V26-RhoD does not show this effect. For clarity reasons the displacement graph of control peroxisomes expressing empty vector is omitted, as it is virtually identical to the control graph in (A).

Intracellular movement of peroxisomes along microtubular tracks is regulated by ATP/LPA receptor co-stimulation and activation of heterotrimeric G proteins. Signaling by both receptor co-stimulation and activation of G proteins caused peroxisomal arrest [2]–[4]. The first clues for a possible involvement of RhoA in this regulation came from LPA signaling that is known to activate RhoA by the trimeric G protein subfamily member G_12/13_ [31], [32]. Therefore, we first tested *C. botulinum* exoenzyme C3 that is known to inactivate Rho proteins of the RhoA subfamily by ADP ribosylation [33]. A significant increase in overall peroxisomal movements compared to untreated cells was observed independent of whether the toxin was added to the culture medium or expressed by transfection (Fig. 1A). As shown by the time series, most peroxisomes moved directionally with frequent changes in direction. Those organelles that did not saltate were at rest for short periods. Following the addition of Nocodazole to exoenzyme C3-treated cells, peroxisomes oscillated, similarly to what could be observed after Nocodazole treatment only (not shown). Both resting and saltating states presupposed association of the organelles to the microtubular cytoskeleton. The motility state elicited by exoenzyme C3 was in sharp contrast to the control state, as the frequency of peroxisomes moving longer distances was significantly increased. On the other hand, transfecting cells with myc-G14V-RhoA, the constitutively active form of RhoA exhibiting remarkably reduced intrinsic GTPase activity and unresponsiveness to RhoA GTPase-activating protein [34], resulted in random oscillations of peroxisomes that were indistinguishable from those observed after Nocodazole treatment (Fig. 1B). This regulatory effect was specific for RhoA. Dominant–active RhoD did not influence peroxisomal motility (Fig. 1B).

**Figure 2:**
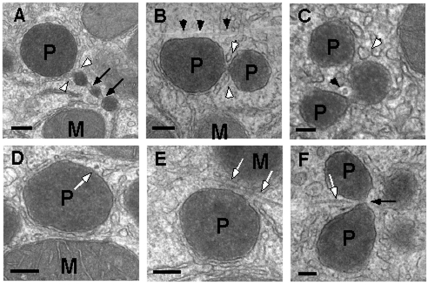
Ultrastructural analysis of peroxisome proliferation in rat liver treated with clofibrate and thyroxin. Peroxisomal constrictions are frequently seen in peroxisomes (P) of different sizes (black arrows in A and F) as well as in peroxisomes associated to microtubular tracks (black arrowheads in B) that sometimes localize close to the constrictions (black arrowhead in C). Filament bundles are running along peroxisomes and close to peroxisomal constrictions (white arrows in D–F) suggesting peroxisome attachment to cytoskeletal filaments. Frequently, cisternae of smooth endoplasmic reticulum (white arrowheads in A–C) are positioned next to the constrictions. M, mitochondrium. The bars represent 200 nm in A, B, D–F and 100 nm in C.

By electron microscopy peroxisomal constrictions were frequently seen preserving membrane continuities between segregated compartments (Fig. 2A–C). Both microtubules (Fig. 2B and C) and microfilament bundles (Fig. 2D–F) were regularly found in close proximity to the organelles (Fig. 2B and C) and smooth ER cisternae possibly delivering Ca^2+^ positioned close to the sites of constrictions. Taken together, these results suggest that peroxisomes contact both microtubules and cytoskeletal filaments. Activated RhoA may uncouple peroxisomes from microtubules as typical microtubule-dependent long-range saltations are no longer observed.

### Peroxisome association to myosin and the actin cytoskeleton

Following incubation of unlabeled rat liver peroxisomes with ^35^S-labeled rat hepatocyte cytosol, proteins with Mr of about 220, 60 and 40 kDa strikingly co-isolated with peroxisomes (not shown). These proteins were identified by nano-HPLC/ESI-MS/MS as non-muscle myosin heavy chain (MyH9) belonging to the class IIA of conventional myosins, α- and β-tubulin and β-actin, respectively. Additional proteins specifically recruiting from cytosol onto peroxisome membranes were detected by extended proteomics analysis. Table 1 lists the proteins identified and related to organelle dynamics and cytoskeletal functions.

**Table 1:** Cytoskeleton and cytoskeleton-related proteins associated with peroxisomes, identified by nano-HPLC/ESI-MS/MS.

Protein	gi accession	MW[kDa]	Sequence coverage [%]
Myosin,heavy polypeptide 9, non-muscle [Rattus norvegicus]	149066032	226,2166	6,70
Tubulin, alpha 1A [Rattus norvegicus]	11560133	50,1036	18,60
Tubulin, beta 2a [Rattus norvegicus]	157819845	49,9209	26,10
Tubulin, alpha 4A [Rattus norvegicus]	55741524	49,8924	13,40
Tubulin, alpha 6 [Rattus norvegicus]	58865558	49,8775	36,30
Tubulin, beta 2b [Rattus norvegicus]	110347600	49,8750	22,70
ATRTC actin beta [Rattus norvegicus]	71620	41,7273	40,50
Actin, beta [Rattus norvegicus]	13592133	41,7000	41,00
Cofilin 1 [Rattus norvegicus]	8393101	18,4280	19,60
14-3-3 zeta/delta [Rattus norvegicus]	62990183	27,7366	10,60

**Figure 3:**
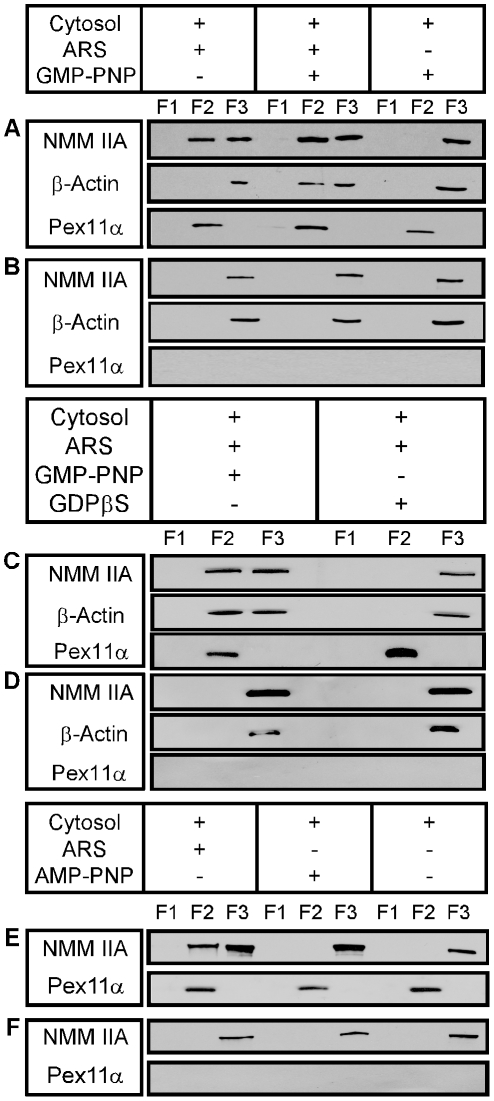
ATP- and GTP-dependent binding of NMM IIA heavy chain and b-actin to isolated rat liver peroxisomes. Organelles were incubated as indicated and floated up in a Nycodenz gradient. Three fractions were recovered from top to bottom (F1–F3). Binding is visualized by immunoblotting (A and C). ATP must be hydrolyzable for binding (E). Peroxisomes are localized in the gradient by the peroxisomal marker Pex11ap. Control incubations lacking peroxisomes do not show floatation of NMM IIA and b-actin (B, D, F) suggesting peroxisomal membranes to be required for binding.

To confirm specific peroxisomal recruitment of these proteins, an *in vitro* peroxisome-binding assay was developed. In this assay peroxisomes were incubated with GMP-PNP-primed cytosol in presence or absence of an ATP-regenerating system and recovered after flotation in a discontinuous Nycodenz gradient. Pex11αp, a peroxisomal membrane marker, floated up in the gradient to fraction F2 just between F1 (250 mM sucrose) and F3 (45% w/v Nycodenz) the latter retaining all the non-floating material. Using antibodies against b-actin and NMM IIA heavy chain, both myosin and actin were detected in the re-isolated peroxisomes. Binding of NMM IIA to peroxisomes required ATP and was observed in the presence of ATP plus GMP-PNP. However, actin recruitment required both nucleotides; the sole presence of GMP-PNP did not mediate recruitment of NMM or actin (Fig. 3A). In control experiments ran in the absence of peroxisomes neither NMM nor actin did float up (Fig. 3B, D, and F). Cytosol preparations contain endogenous guanine nucleotides that in presence of an ATP-regenerating system might become phosphorylated to GTP by nucleotide kinases. Therefore, the nucleotide dependence of NMM IIA and actin recruitment was investigated in presence of GDPγS, a metabolically inert GDP analog. These studies clearly demonstrated that binding of NMM IIA and actin onto peroxisomes requires both ATP and GTP (Fig. 3C). Replacing the ATP-regenerating system for AMP-PNP, a non-hydrolyzable ATP analog, prevented NMM IIA association to peroxisomes suggesting that the association requires hydrolyzable ATP (Fig. 3E).

**Figure 4:**
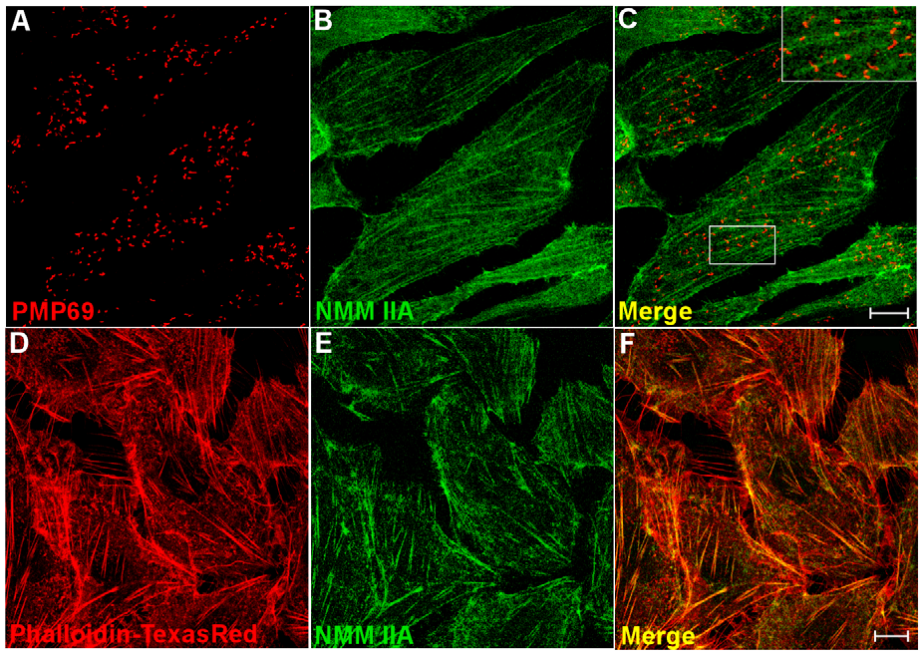
Localization of peroxisomes to acto-myosin complexes in wild type CHO cells. Most peroxisomes visualized by the membrane marker PMP69 (red) are found attached to NMM IIA-associated acto-myosin filaments (A–C). In many cases a number of peroxisomes is aligned along single filaments (see inset) suggesting that peroxisome distribution within the cell is not at random but may be determined by the organelle′s association to acto-myosin filaments (D–F). Images are reproduced from single z-layers. The bar represents 10 mm.

Peroxisomal NMM IIA association was also proved by immunofluorescence. NMM IIA predominantly appeared as filamentous structures bearing peroxisomes closely attached like pearls on a string (Fig. 4A–C and inset in Fig. 4C) rather than having them distributed at random. Analyzing NMM IIA and actin filament stainings revealed nearly complete overlap of both of these structures (Fig. 4D–F). Thus, these immunofluorescence studies strongly suggest peroxisome acto-myosin associations.

### RhoA and its effector ROCKII localize to peroxisomes

**Figure 5:**
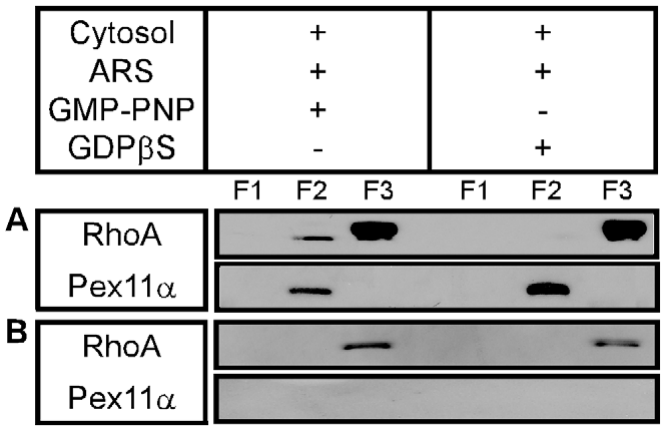
GTP-dependent recruitment of RhoA onto isolated rat liver peroxisomes. No recruitment is seen in the presence of ATP alone (A) and no RhoA is floating up, if peroxisomes are omitted from the incubations (B). Note that without membranes more RhoA precipitates during incubation escaping analysis.

**Figure 6:**
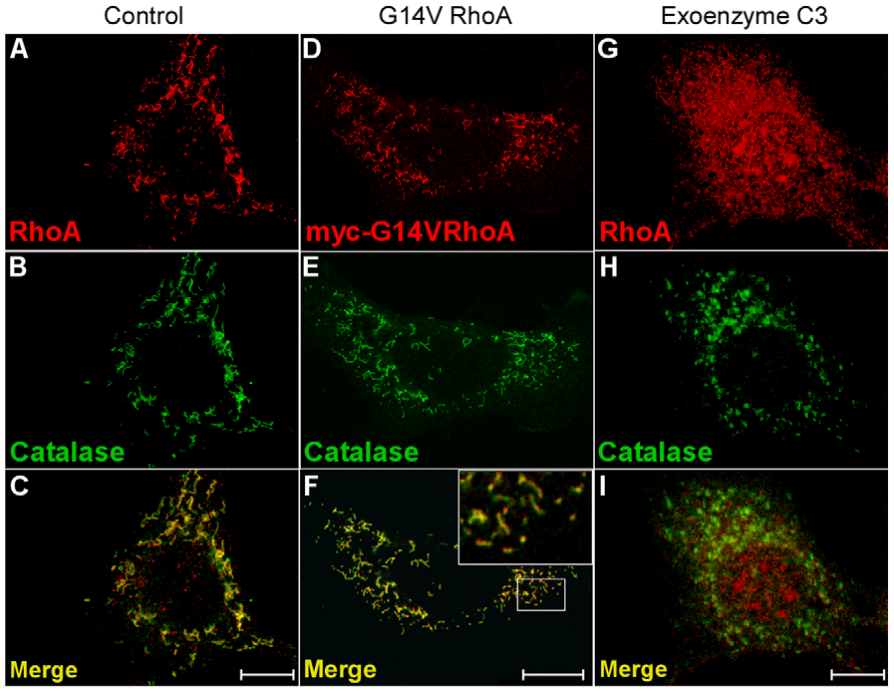
Co-localization of RhoA and peroxisomes in AT3 mouse hepatoma cells. Endogenous (A, C) and transfected dominant-active myc-G14V-RhoA (D, F, red) co-localize to peroxisomes (C, F) visualized by the peroxisomal matrix protein catalase (B, E, green). At higher magnification (inset in F) a patchy distribution of RhoA on peroxisomes becomes apparent. Inactivation of RhoA by *C. botulinum* exoenzyme C3 abolishes RhoA localization to peroxisomes (G–I). Images were reproduced from single z-layers. The bars represent 10 mm.

The GTP dependence of NMM IIA and actin recruitment onto peroxisomes indicate small GTP-binding protein(s) to be involved. Using antibodies specific to RhoA, the GTP-dependent recruitment of RhoA to peroxisomes was observed *in vitro* (Fig. 5). In line with these biochemical assays, we also demonstrated co-localization of peroxisomes and RhoA by transfecting myc-G14VRhoA, a dominant-active mutant of RhoA, into mouse AT3 hepatoma cells (Fig. 6). Whereas both wild type and dominant-active RhoA associated to peroxisomes (Fig. 6A–F), Rho A was not detected on the organelles upon treatment of cell cultures with exoenzyme C3 (Fig. 6G, H, I). By closer examination of the RhoA topology, it appeared that activated RhoA concentrated at distinct sites on the peroxisomal membrane rather than exhibiting a continuous staining pattern (Fig. 6F and inset). In contrast to the RhoA staining, catalase, a peroxisomal matrix protein, constantly labeled the entire organelle. This localization of RhoA to discrete domains of the peroxisomal membrane is reminiscent to the distribution of Pex11αp and PMP69 in CHO cells overexpressing N-terminally myc-tagged Pex11βp [22]. As Pex11βp expression was shown to cause peroxisome proliferation [19], it is tempting to speculate that segregation and concentration of distinct proteins within the peroxisomal membrane and peroxisome proliferation are functionally linked to each other.

**Figure 7:**
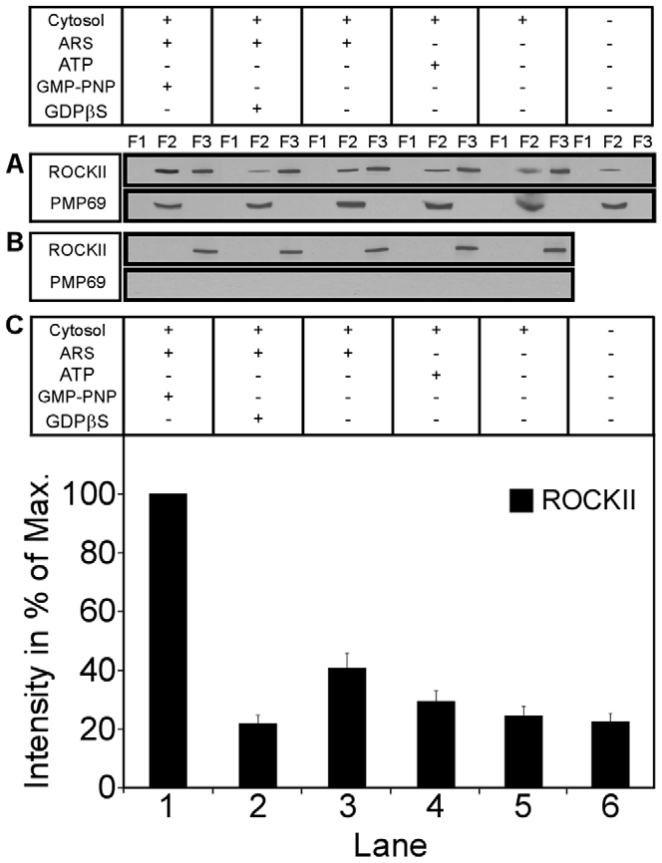
Recruitment of ROCKII onto isolated rat liver peroxisomes. Isolated organelles already have bound small amounts of ROCKII (lane 6 in A). In the presence of ATP and particularly ATP plus GMP-PNP this amount is increased about 2-fold and 5-fold, respectively (lanes 1 and 3 in C). Localization of peroxisomes in the gradient following floatation is indicated by the peroxisomal membrane marker PMP69 (A). Omitting peroxisomes from the incubations abolishes ROCKII floatation (B).

A known effector of RhoA signaling is Rho-associated coiled-coil containing kinase (ROCK). By phosphorylation of myosin light chain (MLC) Rho kinase induces formation of stress fibers and focal adhesions [35]. Two Rho kinase isoforms have been identified, ROCKI and ROCKII. Whereas ROCKI was recently shown to localize to plasma membrane and centrosomes [36], [37], ROCKII appears to be mainly distributed in the cytoplasm and translocates to membranes upon activation by GTP-bound RhoA [38]. Therefore, we focused our attention on ROCKII. Again we used the proxisomal binding assay and observed a significant 3-4-fold increase in the amount of ROCKII recruited from cytosol onto peroxisomes in the presence of ATP plus GMP-PNP (Fig. 7). Interestingly, isolated peroxisomes without being further incubated already had bound ROCKII in low concentration (Fig. 7A and C, lane 6).

**Figure 8:**
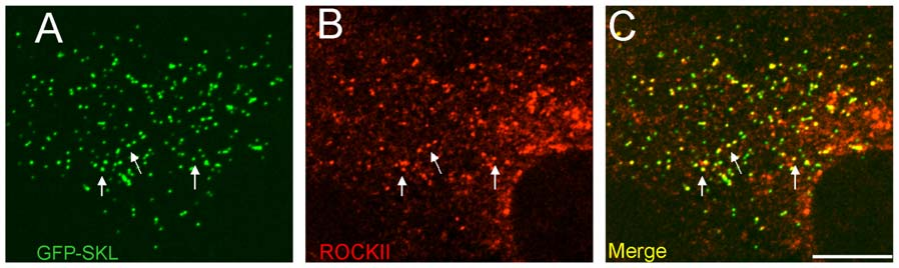
Peroxisomal localization of ROCKII in Huh7 cells. Peroxisomes and ROCKII are visualized by transfecting the cells with GFP-SKL (A, green) and using a ROCKII-specific antibody (B, red), respectively. Merged images (C) show co-localizing structures of which some are indicated by white arrows. Images were assembled from z-projections. The bar represents 10 mm.

We also analyzed peroxisomal attachment of ROCKII by immunofluorescence. Peroxisomes were labeled by GFP carrying a C-terminal peroxisomal targeting signal 1 (GFP-SKL) and endogenous ROCKII was stained using a rabbit polyclonal antiserum (Fig. 8). According to the known broad cellular distribution of ROCKII, cellular background staining was high. Nevertheless, co-localization of ROCKII with the peroxisomal signal was convincingly evident. Quantitative evaluation of 256 GFP-labeled peroxisomes revealed 66 peroxisomes to co-localize with the ROCKII signal, i.e. more than 25% co-localizing structures were recognized.

### Rab8 recruits onto peroxisomes

**Figure 9:**
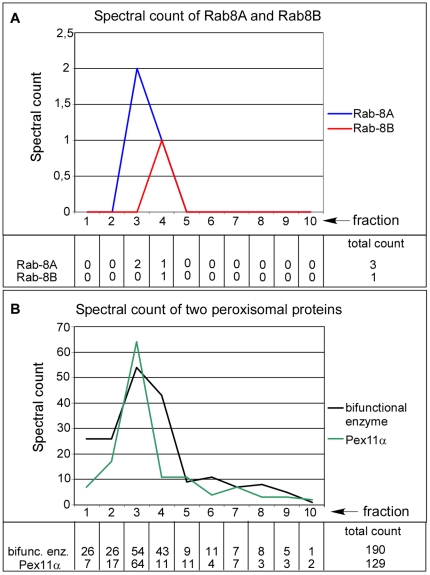
Peroxisomal localization of Rab8 by protein correlation profiling. The post-nuclear fraction of a rat liver homogenate was separated by Nycodenz density gradient centrifugation and the relative abundance of Rab8 (A) and peroxisomal matrix (bifunctional enzyme) and membrane (Pex11ap) marker protein (B)-specific peptides (spectral counts) determined by nano-HPLC/ESI-MS-MS. Peptides specific for Rab8a and Rab8b were exclusively found in fractions 3 and 4 identified as peroxisomal fractions by the marker proteins.

**Figure 10:**
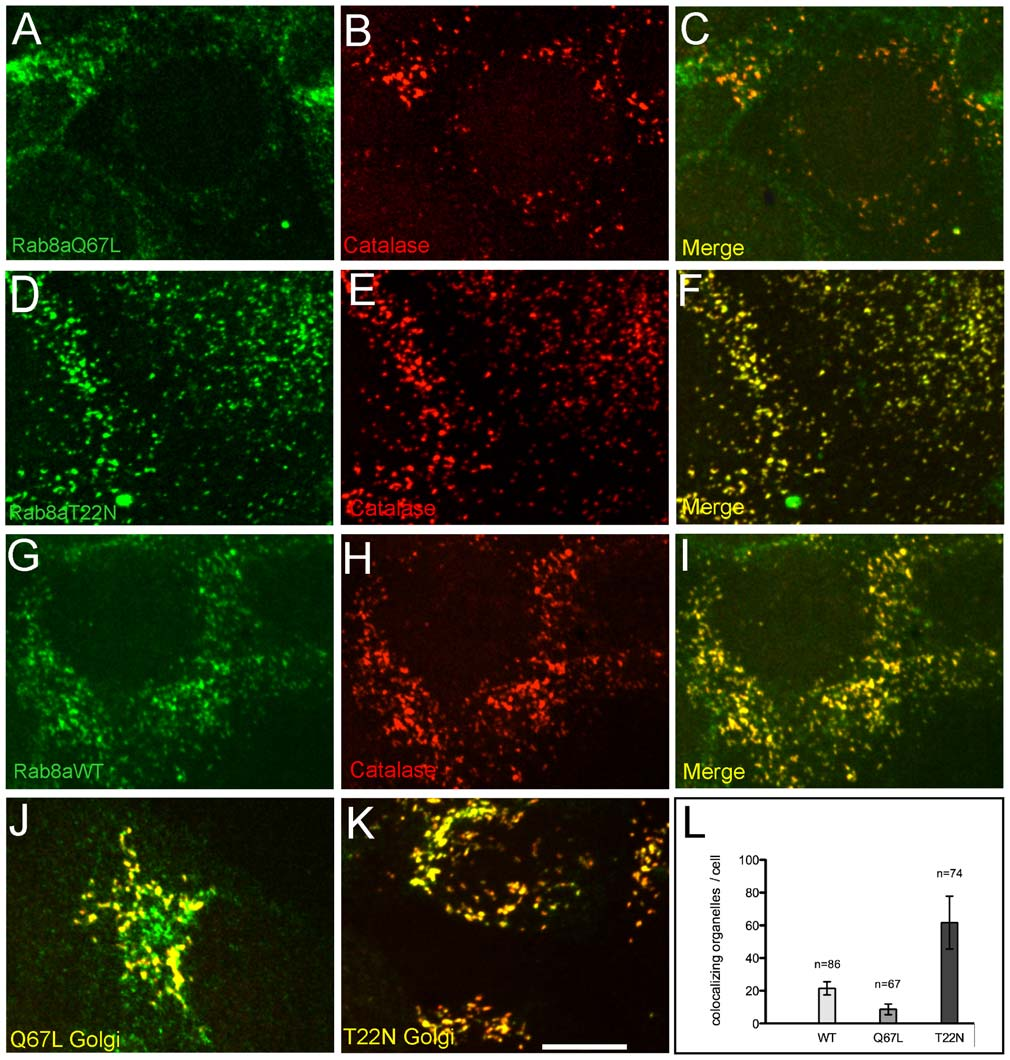
Intracellular localization of Rab8a. Huh7 cells were stably transfected with dominant-active (GFP-Rab8aQ67L; A and J), dominant-inactive (GFP-Rab8aT22N; D and K) and wild type Rab8a (GFP-Rab8aWT; G) constructs. Peroxisomes were visualized by using primary anti-catalase (B, E and H) and Golgi cisternae were stained with primary anti-GM130 (J and K). Alexa FLUOR-labeled secondary antibodies were used for staining. Images were assembled from z-projections. The bar represents 10 mm. The quantitative evaluation of peroxisomes co-localizing with Rab8a following transfection of wild type and mutant Rab8a is shown in (L). n =  number of cells evaluated.

As membrane docking of myosin motors is known to be controlled by Rab proteins [10], [39], [40], we next investigated by an additional proteomics screen the recruitment of small GTPases to the peroxisomal membrane focusing on membrane-associated proteins with M_r_ between 15 and 30 kDa. We detected members of the Rab family and the Rho family as well as ARF, ARF-related and SAR1 proteins. To increase the stringency of our analysis, the Rab proteins were semi-quantitatively profiled based on spectral counts [41] over the entire Nycodenz density gradient separating the post-nuclear fraction of a rat liver homogenate. In this profiling, Rab8a and Rab8b exhibited a gradient distribution strikingly similar to the peroxisomal matrix and membrane markers, bifunctional protein and Pex11ap, respectively (Fig. 9). To further investigate the peroxisomal localization of Rab8, Rab8a was cloned as 5′-GFP fusion construct and dominant-active (Q67L) and dominant-inactive (T22N) forms were stably expressed in Huh7 human hepatocellular carcinoma cells. For co-localization studies, peroxisomes and Golgi were stained with anti-catalase and anti-GM130 antibodies, respectively. The results of these experiments are shown in Fig. 10. (i) Rab8a clearly co-localizes with peroxisomes (Fig. 10A–I) and Golgi cisternae (Fig. 10J and K). (ii) The total average number of peroxisomes per cell changed by expressing wild type GFP-Rab8aWT (54±7; n = 115; Fig. 10G–I), dominant-active Rab8aQ67L (35±6; n = 119; Fig. 10A–C) and dominant-inactive Rab8aT22N (68±15; n = 79; Fig. 10D–F). (iii) Applying organelle-based co-localization (OBCOL) analysis [42], we noted an about tenfold increase in the number of peroxisomes co-localizing with Rab8a in cells transfected with dominant-inactive Rab8aT22N compared with dominant-active Rab8aQ67L (Fig. 10L).

## Discussion

### RhoA signaling regulates peroxisome motility

Our current view of the multiple signaling pathways regulating peroxisome microtubule interaction encompasses both activation of PLA_2_ via G_i_/G_o_ liberating arachidonic acid by ATP and RhoA via G_12_/_13_ by extracellular LPA [3], [4], [31]. Both signals synergize in this process and RhoA appears to be a specific and important intermediate. Most of the activated cytosolic RhoA translocates to cellular membranes leading to saturation of RhoA binding sites and terminating microtubule-peroxisome interactions. Peroxisome oscillations are generated indistinguishable to those following depolymerization of microtubules [43], [44]. On the other hand, exoenzyme C3 rendering RhoA in its inactive GDP-bound form strikingly increased the number of long-range microtubule-based peroxisome saltations. These observations are consistent with the idea that cycling of RhoA between its GDP- and GTP-bound states promotes attachment/detachment cycles of peroxisomes from microtubules. Similar attachment/detachment cycles were reported to affect the motility of pigment granules, Golgi vesicles, lysosomes and mitochondria [45], [46].

### Peroxisome association with the actin cytoskeleton

In previous work evidence has been provided that the actin cytoskeleton obviously is not involved in supporting peroxisome motility. However, a regulated short-range motor protein-based system may be advantageous, as it provides a mechanism tethering organelles at actin-rich intracellular sites and/or exerting mechanical forces [11]. Although the actual mechanism facilitating peroxisome acto-myosin interaction is not known, binding of RhoA-GTP to the peroxisomal membrane may trigger this process possibly by activating NMM IIA. By uncoupling peroxisomes from microtubules, RhoA-GTP may concomitantly activate ROCKII, a serine/threonine kinase acting immediately downstream of RhoA. ROCKII is known to be involved in reorganizing actin structures preventing actin depolymerization. By phosphorylation of the myosin-binding subunit of myosin phosphatase and/or the myosin light chain of myosin II, ROCKII enhances myosin ATPase activity [47], [48]. Moreover, ROCKII contains a well-defined pleckstrin homology domain binding to PtdIns(4,5)P_2_ the synthesis of which in the peroxisomal membrane was recently reported [49].

Myosin might also act in cargo protein assembly at donor membranes, as recently suggested for the biogenesis of melanosomes [50], [51]. Scaffolding of melanosome proteins requires coat and adaptor proteins in addition to myosin. Similar processes may also occur in the peroxisomal system where different to catalase N-myc-tagged Pex11bp [22] and dominant-active RhoA (Fig. 6) both strikingly non-homogenously localize to peroxisomes. Interaction of Pex11βp and RhoA in the peroxisomal membrane and its possible downstream effects on peroxisome biogenesis remain to be established.

### Peroxisomal localization of Rab8

Two Rab8 isotypes have been described, Rab8a and Rab8b. They show distinct tissue distributions. Whereas endogenous Rab8a is predominantly expressed in lung, kidney, skeletal muscle and liver, Rab8b is mainly found in testis, spleen, brain, and heart [52]. The high sequence identity of 83% of both genes might explain why exogenous expression of both isotypes in Huh7 cells show the same subcellular localization and develop rather similar phenotypes. In liver Rab8a might be the predominantly expressed endogenous isotype.

So far Rab8 has been implicated in a variety of functions including constitutive biosynthetic trafficking from the trans-Golgi network and the recycling endosome [53], [54], sorting of apical membrane proteins [55], constitutive and regulated melanosome traffic [56] and regulated ACTH secretion by interacting with TRIP8b [57]. TRIP8b belongs to the tetratricopeptide domain proteins with homology to the peroxisomal targeting signal 1 (PTS1) receptor protein Pex5p known to be involved in peroxisomal matrix protein import [58]–[60]. In vesicle and organelle motility Rab GTPases were implicated in the association of actin motors with the corresponding organellar membranes. Examples are Rab27a and its associated protein MyRIP that mediate melanosome transport by cellular activation of MyoVa and MyoVIIa [61] and Rab11a involved in endosome recycling by linking the endocytic vesicles via Rab11FIP2 to myosin Vb [62] as well as Rab8-activated optineurin recruiting MyoVI [63]. There are still other examples of direct and indirect motor protein effectors of Rab GTPases [54] elucidating multiple ways by which these proteins assure correct transport and vesicle/motor protein interaction.

### Conclusion

Our finding of NMM IIA recruiting onto peroxisomes might be interpreted as actin-dependent intracellular peroxisome transport. However, previous studies addressing this topic did not reveal actin-dependent peroxisome motility [1], [4]. Rather we believe that peroxisomal NMM IIA might locally act in force generation and/or contractile function similar as for example observed in cytokinesis and contractile ring formation [64], [65]. The GTP dependence of this recruitment suggested the involvement of a GTPase. So far several small GTPases were related to peroxisome biogenesis and function. Among these are Rho1p [27], Arf1 and Arf3 in *S. cerevisiae* [26] and mammalian RhoA, Arf1 and Rab8b [4], [26], [57], [58]. In addition, various Rho-GTPases have been implicated in intracellular vesicle trafficking. These include Cdc42 that inhibits recruitment of the microtubular motor dynein to COP I-coated Golgi vesicles [66] and RhoA and Rac affecting both clathrin-dependent [67] and clathrin-independent [68] endocytosis. The latter process was recently reported to involve Arf family GTPases that might be linked to the Rho family via GIT proteins, Arfaptins or ARAPs [69]. Interestingly, mammalian peroxisomes were shown to bind Arf1 and the COP I coat, and in *S.cerevisiae* oleate-induced peroxisome proliferation depends on Arf1 and is strikingly stimulated by deleting Arf3 the ortholog of mammalian Arf6 [26].

In this context the role of Rab8 recruitment to peroxisomes is less clear. Overlap of the Rab8 function with that of Arf6 was reported to occur in the formation of cell protrusions, a process powered by actin polymerization [70], [71]. As both dominant-active and -inactive Rab8 co-localize with peroxisomes and the dominant-inactive form goes along with an increase in peroxisome number, Rab8 seems to occupy a key position on the peroxisomal membrane regulating proliferation.

## Materials and Methods

### Reagents and antibodies

ATP and creatine kinase were from Roche Applied Science (Mannheim), creatine phosphate and exoenzyme C3 from Calbiochem (Frankfurt). Polyclonal rabbit anti-non-muscle myosin IIA and anti-ROCKII were obtained from Sigma (Munich). Monoclonal mouse anti-RhoA (26C4) was from Santa-Cruz Biotechnologies (San Diego) and the monoclonal mouse antibody raised against the c-myc epitope (clone 9E10) from Invitrogen (Karlsruhe). Polyclonal antibodies recognizing peroxisomal proteins were raised in rabbits and were previously described [72]. The expression vector for dominant-active RhoA (pEXV-myc-G14VRhoA) was a gift from Dr. Alan Hall (MRC Laboratory for Molecular Cell Biology, University College, London). Exoenzyme C3 (Sigma, Munich) was added to the cell culture medium at a concentration of 5 mg/ml 24 h prior to analysis. The GFP-Rab8a and GFP-Rab8b constructs were previously described [70]. The vector coding for EGFP-PTS1 was obtained from Clontech (Heidelberg). Other chemicals were from Sigma (Munich).

### Particle-tracking analysis

Time-lapse fluorescence microscopy using a cooled ccd camera was carried out exactly as described [3], [4]. Briefly, time series consisted of 30 pictures taken every 16.5±0.5 s including the 1–2 s time of exposure automatically adjusted according to the fluorescence intensity. Peroxisomal motility was analyzed by animation of the entire time series using the KHOROS software package [73], [74]. Major events of organelle motility were statistically evaluated by subtraction of two consecutive images using the PMIS software (Photometrics, Tucson, AZ, USA) and counting the number of peroxisomes either at rest or in the saltatory or oscillating state. Only the distance covered by the organelles during the time of two consecutive images (16.5±0.5 s) was taken as a measure to determine the number of saltating peroxisomes. Saltations were recognized as being separated by a distance greater than the maximum amplitude of oscillation that was determined in a separate set of experiments and at most corresponded to peroxisomes appearing attached in the overlay of two subsequent time frames [3]. Statistics were based on counting 100 peroxisomes from 4 to 10 cells of 3–7 independent experiments. The displacement histogram was obtained by analyzing complete time series using particle-tracking velocimetry as described [75], [76]. On the x-axis length of translations determined at 0.1 pixel distances was plotted versus frequency (y-axis) in percentage of total organelle counts.

### Peroxisome protein-binding assay

Highly purified peroxisomes were isolated by a combination of rate zonal and Nycodenz equilibrium density gradient centrifugation as previously described [77]. They were obtained in a concentration of 2.5–3.5 mg/ml and stored in small aliquots at −80°C prior to use. Rat liver cytosol was prepared by centrifugation of a rat liver homogenate in buffer H (50 mM Hepes/KOH, pH 7.55, 165 mM KAc, 2 mM MgAc_2_, 1 mM dithiothreitol) supplemented with protease inhibitors (1 mM leupeptin, 10 mM antipain, 100 mM phenylmethylsulfonyl fluoride) [26]. After a final centrifugation at 180,000× g for 90 min (TFT 55.38 rotor, Kontron Instruments, Hanau), the resulting supernatant was concentrated about 10-fold in a MINITAN ultra concentration unit (molecular weight cut-off 10,000; Millipore, Eschborn). The cytosol obtained usually had a final concentration of 45–50 mg/ml. It was stored in aliquots at −80°Cuntil use.

Thawed cytosol (5 mg/ml) was cleared by centrifugation (20,000× g for 20 min at 4°C) and primed by incubation for 30 min at 37°C with either guanosin 5′-(b,-imido)triphosphate (GMP-PNP, 100 µM), or guanosin 5′-(β-thio)diphosphate (GDPγS, 100 µM) or buffer (50 mM HEPES/KOH, pH 7.5). Following centrifugation (200,000× g for 10 min at room temperature) an ATP-regenerating system consisting of 2 mM ATP, 20 mM creatine phosphate, 250 µg/ml creatine kinase in 50 mM HEPES/KOH, pH 7.5 was added. Peroxisomes were freed from Nycodenz by centrifugation and the pelleted peroxisomes (100 mg of protein) resuspended in the cleared cytosol and incubated at 37°C for 30 min. Some incubations contained ATP (2 mM) or AMP-PNP (100 µM) or buffer instead of the ATP-regenerating system. After incubation, organelles were left on ice for 30 min to depolymerize microtubules prior to flotation in a Nycodenz density gradient. To this end, incubated organelles were brought to 45% (w/v) Nycodenz by the addition of 70% (w/v) Nycodenz and overlayed by a layer of 40% (w/v) Nycodenz in sucrose buffer (250 mM sucrose in 10 mM glycylglycine, pH 7.4) followed by a layer of sucrose buffer. Total gradient volume was 600 ml. Centrifugation was carried out at 150,000× g in the SW55II, rotor (Beckman, Krefeld) for 3 h at 4°C. Three fractions indicated as F1, F2 and F3 were collected from top to bottom.

Proteins from reisolated peroxisomes were separated by SDS-PAGE using pre-cast Tris-Tricine 10–20% gradient gels (Anamed, Bad Ems) and subsequently transferred onto PVDF-membranes (Pall, Bad Kreuznach) for 2 hours at 400 mA. Proteins were visualized by enhanced chemiluminescence.

^35^S-labeled cytosol was isolated from metabolically labelled primary rat hepatocytes grown in DMEM/Hanks'F12 (1∶1) containing 10% FCS, penicillin/streptomycin, 1% L-glutamine, 0.5 µM insulin, 1 µM dexamethasone and 1 µM triiodothyronine over night at 37°C and 5% CO_2_. For radioactive labelling, cells were incubated for 3 h in methionine/cysteine-free DMEM supplemented with 10 mM HEPES/NaOH, pH 7.4, 10% dialysed FCS, penicillin/streptomycin, 1% L-glutamine, 0.5 µM insulin, 1 µM dexamethasone and 1 µM triiodotyronine in the presence of 37.5 mCi/ml of ^35^S-methionine/cysteine (>1000 Ci/mmol, GE-Healthcare, Braunschweig). Cells were homogenized on ice in buffer H and the post-nuclear supernatant was centrifuged at 100,000× g in the TLA45 rotor (Beckman, Krefeld) for 1.5 hours at 4°C. Specific activity of the obtained ^35^S-labeled cytosol was 45 mCi/mg of protein.

### Autoradiography

Peroxisomes were incubated as described above using metabolically labelled rat liver cytosol in the presence of 2 mM ATP, 100 µM GTPγS and phosphatase inhibitor cocktail 2 (Sigma-Aldrich). Peroxisomes were separated from the bulk of cytosol by Nycodenz-density gradient flotation through a layer of 43% (w/v) Nycodenz and the peroxisomal proteins subjected to SDS-PAGE. To visualize ^35^S-labelled proteins, gels were dried on Whatman 3 MM paper and exposed to X-ray films (BioMax MR Scientific Imaging, Berlin).

### Proteomics analyses of peroxisomes and peroxisomal membranes

Proteins of the reisolated peroxisomes visualized by autoradiography or by SDS-PAGE were identified by nano-HPLC/ESI-MS/MS [78]. Tryptic peptide samples were analyzed on a Dionex LC Packings HPLC system (Dionex LC Packings, Idstein) directly coupled to a Bruker Daltronics HCT plus ion trap instrument equipped with nanoelectrospray ion source (Bruker, Bremen) and distal coated SilicaTips (New Objective, Wobern, MA, USA). For profiling of proteins associated with peroxisomal membranes, Nycodenz gradient fractions number 1 to 10 were individually treated with NaCO_3_, pH 11.5. The resulting membrane pellets were then subjected to SDS-PAGE and proteins stained by colloidal Coomassie Brilliant Blue G-250. For analysis of the low-molecular weight (<30 kDa) region the gel was equally cut into three slices and proteins were digested in-gel with trypsin and further subjected to proteomics analysis as described [78]. Briefly, nano-HPLC/ESI-MS/MS analyses were performed on a 7-Tesla Finnigan LTQ-FT (Thermo Electron, Bremen) instrument equipped with a nanoelectrospray ion source. Samples were analyzed in a data-dependent mode fragmenting the three most intense ions in the linear ion trap by low-energy CID and accurately performing in parallel mass measurements in the FTICR cell.

For peptide and protein identification, MS/MS datasets were generally correlated with the rat International Protein Index (IPI; www.ebi.ac.uk) database using the MASCOT algorithm. Proteins were identified based on at least one unique peptide and a false positive rate below 5%. To semi-quantitatively follow proteins of interest across the density gradient fractions, the respective spectral counts were calculated [41] and plotted against the number of gradient fractions in order to generate protein profiles.

### Transfection and immunofluorescence

CHO-K1 and Huh7 cells were grown in DMEM containing 10% FCS, penicillin/streptomycin and 1% L-glutamine, whereas AT3F mouse hepatoma cells were maintained in DMEM/Hanks F12 (1∶1) supplemented with 10% FCS, penicillin/streptomycin, 1% L-glutamine, 0.5 µM insulin, 1 µM dexamethasone, 1 µM triiodotyronine at 37°C and 5% CO_2_. Cells stably expressing EGFP—SKL were prepared as previously described [3], [4]. Transient expression of myc-G14V-RhoA, a dominant-active mutant of RhoA [79] was achieved by transfection using the Nucleofector II device (Lonza, Basel) and an optimized protocol for mouse hepatocytes. For exoenzyme C3 treatment, the toxin (5 mg/ml) either was added to the medium two days prior to analysis or was expressed as a myc-tagged protein by transfection [48]. Transfection of GFP-Rab8a and GFP-Rab8b in Huh7 cells was done using calcium phosphate. Cells were selected with 400 mg/l Geneticin for stable transfection and subsequently maintained in medium containing 250 mg/l Geneticin. For immunofluorescence, cells were grown on glass cover slips, fixed in 3% PFA and permeabilized with 1% TX-100 for 5 min at room temperature. After blocking with 10% BSA, cells were incubated with primary antibody for 1 h at 37°C followed by the appropriate FITC-, TRITC- or Alexa FLUOR-labeled secondary antibody (Invitrogen, Karlsruhe). Cover slips were mounted using Mowiol/p-phenylendiamine 10∶1 in 0.1 M Tris/HCl, pH 8.7 (Sigma, Munich) or VectaShield hard set mounting medium (Vector Laboratories). Microscopy was carried out using the confocal laser-scanning microscope LSM Meta 510 (Zeiss, Jena) or an Axio Oberserver SD confocal microscope (Zeiss, Göttingen).

### Colocalization analysis

Prior to quantification of co-localizing structures, image stacks were filtered using the Gauss filter implemented in the image aquisition software (Axiovision 4.8, Zeiss, Göttingen) to remove noise. Stacks were subsequently exported as 8-bit greyscale images in tif-format and the analysis was performed using ImageJ (http://rsb.info.nih.gov/ij/) and the OBCOL plugin [42]. The OBCOL plugin represents an automated pipeline for structure segmentation/aggregation into 3D organelles and subsequent statistical evaluation. OBCOL parameters were set to default of the OBCOL pipeline, a Pearson coefficient of ≥0,5 indicated co-localization. For counting the total number of organelles, the „nucleus counter“ plugin of ImageJ was used.

### Electron microscopy

To generate peroxisome proliferating conditions, rats weighing about 150 g were treated with clofibrate (0.5% in the diet) and daily s.c. injections of 100 mg T_4_ for five consecutive days. Following anesthesia rats were fixed by transcardial perfusion with 2.5% glutaraldehyde in 0.1 M Na-cacodylate buffer, pH 7.6 containing 2% polyvinylpyrrolidone and 0.05% CaCl_2_. Microslicer liver sections (60–80 mm thick) were incubated in alkaline diaminobenzidine (pH 10.0) for 30 min. Samples were postfixed by cacodylate-buffered 1% osmium tetroxide for 1 h and stained en bloc in 1% uranyl acetate for 30 min. After dehydration in graded ethanol samples were embedded in Epon. Ultrathin sections were stained with lead citrate and analyzed using a Zeiss EM 906E [80].

## References

[1] Rapp S, Saffrich R, Anton M, Jakle U, Ansorge W (1996). Microtubule-based peroxisome movement.. J Cell Sci.

[2] Wiemer EA, Wenzel T, Deerinck TJ, Ellisman MH, Subramani S (1997). Visualization of the peroxisomal compartment in living mammalian cells: dynamic behavior and association with microtubules.. J Cell Biol.

[3] Huber C, Saffrich R, Anton M, Passreiter M, Ansorge W (1997). A heterotrimeric G protein-phospholipase A2 signaling cascade is involved in the regulation of peroxisomal motility in CHO cells.. J Cell Sci.

[4] Huber CM, Saffrich R, Ansorge W, Just WW (1999). Receptor-mediated regulation of peroxisomal motility in CHO and endothelial cells.. EMBO J.

[5] Huber CM, Saffrich R, Gorgas K, Just WW (1999). Organelle motility regulated by the cell's environment: dissection of signaling pathways regulating movements of peroxisomes.. Protoplasma.

[6] Huber C (1999). Regulation der Bewegung von Peroxisomen..

[7] Mathur J, Mathur N, Hulskamp M (2002). Simultaneous visualization of peroxisomes and cytoskeletal elements reveals actin and not microtubule-based peroxisome motility in plants.. Plant Physiol.

[8] Fagarasanu A, Fagarasanu M, Rachubinski RA (2007). Maintaining peroxisome populations: a story of division and inheritance.. Annu Rev Cell Dev Biol.

[9] Hoepfner D, van den Berg M, Philippsen P, Tabak HF, Hettema EH (2001). A role for Vps1p, actin, and the Myo2p motor in peroxisome abundance and inheritance in Saccharomyces cerevisiae.. J Cell Biol.

[10] Seabra MC, Coudrier E (2004). Rab GTPases and myosin motors in organelle motility.. Traffic.

[11] Soldati T, Schliwa M (2006). Powering membrane traffic in endocytosis and recycling.. Nature Rev.

[12] Orth JD, Krueger EW, Cao H, McNiven MA (2002). The large GTPase dynamin regulates actin comet formation and movement in living cells.. Proc Natl Acad Sci U S A.

[13] Southwick FS, Li W, Zhang F, Zeile WL, Purich DL (2003). Actin-based endosome and phagosome rocketing in macrophages: activation by the secretagogue antagonists lanthanum and zinc.. Cell Mot Cytoskeleton.

[14] Buss F, Luzio JP, Kendrick-Jones J (2002). Myosin VI, an actin motor for membrane traffic and cell migration.. Traffic.

[15] Ikonen E, de Almeid JB, Fath KR, Burgess DR, Ashman K (1997). Myosin II is associated with Golgi membranes: identification of p200 as nonmuscle myosin II on Golgi-derived vesicles.. J Cell Sci.

[16] Wu X, Bowers B, Rao K, Wei Q, Hammer JA (1998). Visualization of melanosome dynamics within wild-type and dilute melanocytes suggests a paradigm for myosin V function In vivo.. J Cell Biol.

[17] De Vos KJ, Allan VJ, Grierson AJ, Sheetz MP (2005). Mitochondrial function and actin regulate dynamin-related protein 1-dependent mitochondrial fission.. Curr Biol.

[18] Koch A, Thiemann M, Grabenbauer M, Yoon Y, McNiven MA (2003). Dynamin-like protein 1 is involved in peroxisomal fission.. J Biol Chem.

[19] Li X, Gould SJ (2003). The dynamin-like GTPase DLP1 is essential for peroxisome division and is recruited to peroxisomes in part by PEX11.. J Biol Chem.

[20] Yan M, Rayapuram N, Subramani S (2005). The control of peroxisome number and size during division and proliferation.. Curr Opin Cell Biol.

[21] McNiven MA, Cao H, Pitts KR, Yoon Y (2000). The dynamin family of mechanoenzymes: pinching in new places.. TIBS.

[22] Lay D, Gorgas K, Just WW (2006). Peroxisome biogenesis: where Arf and coatomer might be involved.. Biochim Biophys Acta.

[23] Passreiter M, Anton M, Lay D, Frank R, Harter C (1998). Peroxisome biogenesis: involvement of ARF and coatomer.. J Cell Biol.

[24] Takai Y, Sasaki T, Matozaki T (2001). Small GTP-binding proteins.. Physiol Rev.

[25] Grosshans BL, Ortiz D, Novick P (2006). Rabs and their effectors: achieving specificity in membrane traffic.. Proc Natl Acad Sci U S A.

[26] Lay D, Grossans L, Heid H, Gorgas K, Just WW (2005). Binding and functions of ADPribosylation factor on mammalian and yeast peroxisomes.. J Biol Chem.

[27] Marelli M, Smith JJ, Jung S, Yi E, Nesvizhskii AI (2004). Quantitative mass spectrometry reveals a role for the GTPase Rho1p in actin organization on the peroxisome membrane.. J Cell Biol.

[28] Rottensteiner H, Stein K, Sonnenhol E, Erdmann R (2003). Conserved function of pex11p and the novel pex25p and pex27p in peroxisome biogenesis.. Mol Biol Cell.

[29] Tam YY, Torres-Guzman JC, Vizeacoumar FJ, Smith JJ, Marelli M (2003). Pex11-related proteins in peroxisome dynamics: a role for the novel peroxin Pex27p in controlling peroxisome size and number in Saccharomyces cerevisiae.. Mol Biol Cell.

[30] Thoms S, Erdmann R (2005). Dynamin-related proteins and Pex11 proteins in peroxisome division and proliferation.. FEBS J.

[31] Moolenaar WH, van Meeteren LA, Giepmans BN (2004). The ins and outs of lysophosphatidic acid signaling.. Bioessays.

[32] Rumenapp U, Blomquist A, Schworer G, Schablowski H, Psoma A (1999). Rho-specific binding and guanine nucleotide exchange catalysis by KIAA0380, a dbl family member.. FEBS Lett.

[33] Fritz G, Aktories K (1994). ADP-ribosylation of Rho proteins by Clostridium botulinum exoenzyme C3 is influenced by phosphorylation of Rho-associated factors.. Biochem J.

[34] Garrett MD, Self AJ, van Oers C, Hall A (1989). Identification of distinct cytoplasmic targets for ras/R-ras and rho regulatory proteins.. J Biol Chem.

[35] Kawano Y, Fukata Y, Oshiro N, Amano M, Nakamura T (1999). Phosphorylation of myosin-binding subunit (MBS) of myosin phosphatase by Rhokinase in vivo.. J Cell Biol.

[36] Chevrier V, Piel M, Collomb N, Saoudi Y, Frank R (2002). The Rho-associated protein kinase p160ROCK is required for centrosome positioning.. J Cell Biol.

[37] Pinner S, Sahai E (2008). PDK1 regulates cancer cell motility by antagonising inhibition of ROCK1 by RhoE.. Nature Cell Biol.

[38] Noma K, Oyama N, Liao JK (2006). Physiological role of ROCKs in the cardiovascular system.. Am J Physiol.

[39] Hume AN, Collinson LM, Rapak A, Gomes AQ, Hopkins CR (2001). Rab27a regulates the peripheral distribution of melanosomes in melanocytes.. J Cell Biol.

[40] Wu X, Rao K, Bowers MB, Copeland NG, Jenkins NA (2001). Rab27a enables myosin Va-dependent melanosome capture by recruiting the myosin to the organelle.. J Cell Sci.

[41] Liu H, Sadygov RG, Yates JR (2004). A model for random sampling and estimation of relative protein abundance in shotgun proteomics.. Anal Chem.

[42] Woodcroft BJ, Hammond L, Stow JL, Hamilton NA (2009). Automated organelle-based colocalization in whole-cell imaging.. Cytometry A.

[43] Boukharov AA, Cohen CM (1998). Guanine nucleotide-dependent translocation of RhoA from cytosol to high affinity membrane binding sites in human erythrocytes.. Biochem J.

[44] Kranenburg O, Poland M, van Horck FP, Drechsel D, Hall A (1999). Activation of RhoA by lysophosphatidic acid and Galpha12/13 subunits in neuronal cells: induction of neurite retraction.. Mol Biol Cell.

[45] Harada A, Takei Y, Kanai Y, Tanaka Y, Nonaka S (1998). Golgi vesiculation and lysosome dispersion in cells lacking cytoplasmic dynein.. J Cell Biol.

[46] Tanaka Y, Kanai Y, Okada Y, Nonaka S, Takeda S (1998). Targeted disruption of mouse conventional kinesin heavy chain, kif5B, results in abnormal perinuclear clustering of mitochondria.. Cell.

[47] Fukata Y, Amano M, Kaibuchi K (2001). Rho-Rho-kinase pathway in smooth muscle contraction and cytoskeletal reorganization of non-muscle cells.. Trends Pharmacol Sci.

[48] Hall A (1998). Rho GTPases and the actin cytoskeleton.. Science.

[49] Jeynov B, Lay D, Schmidt F, Tahirovic S, Just WW (2006). Phosphoinositide synthesis and degradation in isolated rat liver peroxisomes.. FEBS Lett.

[50] Coudrier E (2007). Myosins in melanocytes: to move or not to move?. Pigment Cell Res.

[51] Sorkin A (2004). Cargo recognition during clathrin-mediated endocytosis: a team effort.. Cur Opin Cell Biol.

[52] Armstrong J, Thompson N, Squire JH, Smith J, Hayes B (1996). Identification of a novel member of the Rab8 family from the rat basophilic leukaemia cell line, RBL.2H3.. J Cell Sci.

[53] Knodler A, Feng S, Zhang J, Zhang X, Das A (2010). Coordination of Rab8 and Rab11 in primary ciliogenesis.. Proc Natl Acad Sci U S A.

[54] Stenmark H (2009). Rab GTPases as coordinators of vesicle traffic.. Nature Rev.

[55] Sato T, Mushiake S, Kato Y, Sato K, Sato M (2007). The Rab8 GTPase regulates apical protein localization in intestinal cells.. Nature.

[56] Chakraborty AK, Funasaka Y, Araki K, Horikawa T, Ichihashi M (2003). Evidence that the small GTPase Rab8 is involved in melanosome traffic and dendrite extension in B16 melanoma cells.. Cell Tissue Res.

[57] Chen S, Liang MC, Chia JN, Ngsee JK, Ting AE (2001). Rab8b and its interacting partner TRIP8b are involved in regulated secretion in AtT20 cells.. J Biol Chem.

[58] Fransen M, Amery L, Hartig A, Brees C, Rabijns A (2008). Comparison of the PTS1- and Rab8b-binding properties of Pex5p and Pex5Rp/TRIP8b.. Biochim Biophys Acta.

[59] Ma C, Subramani S (2009). Peroxisome matrix and membrane protein biogenesis.. IUBMB Life.

[60] Meinecke M, Cizmowski C, Schliebs W, Kruger V, Beck S (2010). The peroxisomal importomer constitutes a large and highly dynamic pore.. Nature Cell Biol.

[61] Ramalho JS, Lopes VS, Tarafder AK, Seabra MC, Hume AN (2009). Myrip uses distinct domains in the cellular activation of myosin VA and myosin VIIA in melanosome transport.. Pigment Cell Melanoma Res.

[62] Hales CM, Vaerman JP, Goldenring JR (2002). Rab11 family interacting protein 2 associates with Myosin Vb and regulates plasma membrane recycling.. J Biol Chem.

[63] Sahlender DA, Roberts RC, Arden SD, Spudich G, Taylor MJ (2005). Optineurin links myosin VI to the Golgi complex and is involved in Golgi organization and exocytosis.. J Cell Biol.

[64] Even-Ram S, Doyle AD, Conti MA, Matsumoto K, Adelstein RS (2007). Myosin IIA regulates cell motility and actomyosin-microtubule crosstalk.. Nature Cell Biol.

[65] Yumura S (2001). Myosin II dynamics and cortical flow during contractile ring formation in Dictyostelium cells.. J Cell Biol.

[66] Chen JL, Fucini RV, Lacomis L, Erdjument-Bromage H, Tempst P (2005). Coatomer-bound Cdc42 regulates dynein recruitment to COPI vesicles.. J Cell Biol.

[67] Qualmann B, Mellor H (2003). Regulation of endocytic traffic by Rho GTPases.. Biochem J.

[68] Nie Z, Randazzo PA (2006). Arf GAPs and membrane traffic.. J Cell Sci.

[69] Ridley AJ (2006). Rho GTPases and actin dynamics in membrane protrusions and vesicle trafficking.. TIBS.

[70] Hattula K, Furuhjelm J, Tikkanen J, Tanhuanpaa K, Laakkonen P (2006). Characterization of the Rab8-specific membrane traffic route linked to protrusion formation.. J Cell Sci.

[71] Mejillano MR, Kojima S, Applewhite DA, Gertler FB, Svitkina TM (2004). Lamellipodial versus filopodial mode of the actin nanomachinery: pivotal role of the filament barbed end.. Cell.

[72] Koster A, Heisig M, Heinrich PC, Just WW (1986). In vitro synthesis of peroxisomal membrane polypeptides.. Biochem Biophys Res Commun.

[73] Herr S, Bastian T, Pepperkok R, Boulin C, Ansorge W (1993). A fully automated image acquisition and analysis system for low light level fluorescence microscopy.. Meth Mol Cell Biol.

[74] Rasure J, Williams C, Argiro D, Sauer T (1990). A visual language and software development environment for image processing.. Int J Imaging Systems Technol.

[75] Hering F, Leue C, Wierzimok D, Jähne B (1997). Particle tracking velocimetry beneath water waves. Part I: visualization and tracking algorithms.. Exp Fluids.

[76] Tvarusko W, Bentele M, Misteli T, Rudolf R, Kaether C (1999). Time-resolved analysis and visualization of dynamic processes in living cells.. Proc Natl Acad Sci U S A.

[77] Hartl FU, Just WW, Koster A, Schimassek H (1985). Improved isolation and purification of rat liver peroxisomes by combined rate zonal and equilibrium density centrifugation.. Arch Biochem Biophys.

[78] Wiese S, Gronemeyer T, Ofman R, Kunze M, Grou CP (2007). Proteomics characterization of mouse kidney peroxisomes by tandem mass spectrometry and protein correlation profiling.. Mol Cell Proteomics.

[79] Adamson P, Paterson HF, Hall A (1992). Intracellular localization of the P21rho proteins.. J Cell Biol.

[80] Gorgas K, Krisans SK (1989). Zonal heterogeneity of peroxisome proliferation and morphology in rat liver after gemfibrozil treatment.. J Lipid Res.

